# Prediction of hepatocellular carcinoma using age and liver stiffness on transient elastography after hepatitis C virus eradication

**DOI:** 10.1038/s41598-022-05492-5

**Published:** 2022-01-27

**Authors:** Masato Nakai, Yoshiya Yamamoto, Masaru Baba, Goki Suda, Akinori Kubo, Yoshimasa Tokuchi, Takashi Kitagataya, Ren Yamada, Taku Shigesawa, Kazuharu Suzuki, Akihisa Nakamura, Takuya Sho, Kenichi Morikawa, Koji Ogawa, Ken Furuya, Naoya Sakamoto

**Affiliations:** 1 Department of Gastroenterology and Hepatology, Graduate School of Medicine, Hokkaido University, North 15, West 7, Kita-ku, Sapporo, Hokkaido 060-8638 Japan; 2 Department of Gastroenterology, Hakodate Municipal Hospital, Hakodate, Hokkaido Japan; 3 Department of Gastroenterology, JCHO Hokkaido Hospital, Sapporo, Hokkaido Japan

**Keywords:** Hepatitis C, Hepatocellular carcinoma

## Abstract

Liver stiffness measurement (LSM) is a useful tool for assessing advanced liver fibrosis, an important risk factor for hepatocellular carcinoma (HCC) following hepatitis C (HCV) eradication. This study aimed to clarify the non-invasive factors associated with HCC following sustained virological response (SVR) and to identify the low-risk group. 567 patients without history of HCC who achieved SVR at 24 weeks (SVR24) after IFN-free treatment were retrospectively analyzed. The cumulative incidence of HCC and the risk factors were examined using pre-treatment and SVR24 data. The median observation period was 50.2 months. Thirty cases of HCC were observed, and the 4-year cumulative incidence of HCC was 5.9%. In multivariate analysis, significant pre-treatment factors were age ≥ 71 years (hazard ratio [HR]: 3.402) and LSM ≥ 9.2 kPa (HR: 6.328); SVR24 factors were age ≥ 71 years (HR: 2.689) and LSM ≥ 8.4 kPa (HR: 6.642). In cases with age < 71 years and LSM < 8.4 kPa at the time of SVR24, the 4-year cumulative incidence of HCC was as low as 1.1%. Both pre-treatment LSM (≥ 9.2 kPa) and SVR24 LSM (≥ 8.4 kPa) and age (≥ 71 years) are useful in predicting the risk of HCC after SVR with IFN-free treatment. Identification of low-risk individuals may improve the efficiency of follow-up.

## Introduction

Hepatitis C is an important global health problem, with approximately 71 million people chronically infected with this disease globally in 2017^1^. In chronic infection of hepatitis C virus (HCV), liver fibrosis gradually progresses over time, resulting in a high rate of liver cirrhosis and hepatocellular carcinoma (HCC). Approximately 840,000 new cases of HCC occur annually, and it is the fourth leading cause of cancer-related deaths globally^2^. Therefore, until recently, interferon (IFN)-based antiviral therapies were used to eradicate HCV, suppress the progression of liver fibrosis, and suppress the development of HCC. Direct-acting antivirals (DAAs) became available in 2011, and IFN-free treatment with only DAAs is now commonly used. The achievement rate of sustained virological response (SVR) using IFN-free treatment is significantly high (approximately 95%), and it has become possible to achieve virus elimination in almost all cases. However, it is known that HCC is often observed even in cases of HCV eradication.

Various factors have been reported to predict the occurrence of HCC after HCV eradication using both IFN-based and IFN-free treatment. Especially in IFN-based treatment, advanced liver fibrosis is known to be an important risk factor for HCC^3–15^. Liver biopsy is the gold standard for diagnosing liver fibrosis; however, it is an invasive procedure. Therefore, currently, it is less common to evaluate histological liver fibrosis using liver biopsy before starting DAA treatment. Alternatively, ultrasonic transient elastography^16^, a non-invasive method for diagnosing fibrosis, is often used. Using this method, quantitative liver stiffness measurement (LSM) can be easily evaluated. Analyses of the associations between LSM at pre-DAA and post-DAA with the occurrence of HCC after HCV eradication by DAAs are still limited.

This study aimed to clarify the non-invasive factors associated with the occurrence of HCC after treatment of HCV infection with IFN-free DAAs. Additionally, using the extracted factors, we aimed to identify patients with a low or a high risk based on HCC after successful HCV eradication using IFN-free DAAs and clarify their usefulness in HCC surveillance.

## Results

### Patient characteristics and cumulative incidence of HCC

Overall, the median age of 567 patients who achieved SVR24 was 66 years, and 259 patients were men (45.7%) (Table 1). Additionally, 77 (13.6%) patients were clinically diagnosed with cirrhosis. The overall median body mass index was 22.8 kg/m^2^, and diabetes was observed in 105 (18.5%) patients. Regarding HCV genotype, 359 (63.3%) patients and 195 (34.4%) patients had type 1 and type 2 genotypes, respectively. The treatments used were daclatasvir (DCV) + asunarprevier (ASV) in 98 (17.3%) patients, sofosbuvir/ledipasvir (SOF/LDV) in 153 (27.0%) patients, ombitasvir/paritaprevir/ritonavir in 30 (5.3%) patients, elbasvir + grazoprevir in 33 (5.8%) patients, DCV + ASV + beclabuvir in 5 (0.9%) patients, glecaprevir/pibrentasvir in 91 (16.0%) patients, SOF + ribavirin (RBV) in 145 (25.6%) patients, SOF/velpatasvir in 3 (0.5%) patients, SOF/LDV + RBV in 7 (1.2%) patients, and others in 2 (0.4%) patients. The median blood biochemical data before treatment were the following: platelet count, 16.5 × 10^4^/µL; aspartate transaminase, 40 IU/L; alanine aminotransferase (ALT), 38 IU/L; albumin, 4.1 g/dL; α-fetoprotein (AFP), 4.2 ng/mL; and Fibrosis-4 index (FIB4 index), 2.72. The median liver stiffness on ultrasonic elastography before treatment was 7.1 kPa, and the median observation period from the start of HCV treatment was 50.2 months.

**Table 1:** Baseline characteristics of patients.

Number of patients	567
Sex, Male n(%)	259 (45.7)
Age (years old)	66 (16–92)
BMI (kg/m^2^)^†^	22.8 (12.8–46.5)
Liver cirrhosis n(%)	77 (13.6)
HCV genotype (1/2/Mix/Others/unknown)	359/195/7/4/2
Diabetes, n(%)	105 (18.5)
Therapy resume n(%)	
DCV + ASV	98 (17.3)
SOF/LDV	153 (27.0)
OMV/PTV/r	30 (5.3)
EBR + GZR	33 (5.8)
DCV + ASV + BCV	5 (0.9)
GLE/PIB	91 (16.0)
SOF + RBV	145 (25.6)
SOF/VEL	3 (0.5)
SOF/LDV + RBV	7 (1.2)
Others	2 (0.4)
Platelet (× 10^4^/µl)	16.5 (0.7–41.3)
AST (IU/L)	40 (10–342)
ALT (IU/L)	38 (5–389)
GGT (IU/L)^‡^	33 (8–777)
Albumin (g/dl)	4.1 (2.4–5.0)
AFP (ng/ml)	4.2 (1.0–250.8)
Type4 collagen 7S (ng/ml)^§^	4.8 (1.5–17.0)
Hyaluronic acid (ng/ml)^¶^	103 (3.2–6660)
WFA^ +^ Mac-2 Binding protein (C.O.I)^††^	1.81 (0.19–20.0)
FIB4 index	2.72 (0.29–82.8)
LSM (kPa)	7.1 (2.9–66.4)
CAP (db/m)^‡‡^	219 (100–486)
Median obsevation period from start of treatment (Months)	50.2 (7–92)

^†^ The data include only 441 patients, ^‡^ The data include only 355 patients.

^§^ The data include only 233 patients, ^¶^The data include only 327 patients.

^††^ The data include only 431 patients, ^‡‡^ The data include only 332 patients.

*BMI* Body mass index, *HCV* Hepatitis C virus, *DCV* daclatasvir, *ASV* asnerprevier, *SOF/LDV* sofosbuvir/ledipasvir, *OMV/PTV/r* ombitasvir/paritaprevir/ritonavir, *EBR* Elbasvir, *GZR* Grazoprevir, *BCV* Beclabuvir, *GLE/PIB* Glecaprevir/Pibrentasvir, *RBV* Ribavirin, *SOF/VEL* sofosbuvir/velpatasvir, *AST* Aspartate transaminase, *ALT* Alanine aminotransferase, *GGT* γ-glutamyltransferase, *AFP* α-fetoprotein, *WFA* Wisteria floribunda agglutinin, *LSM* Liver stiffness measurement, *CAP* Controlled attenuation Parameter.

**Figure 1:**
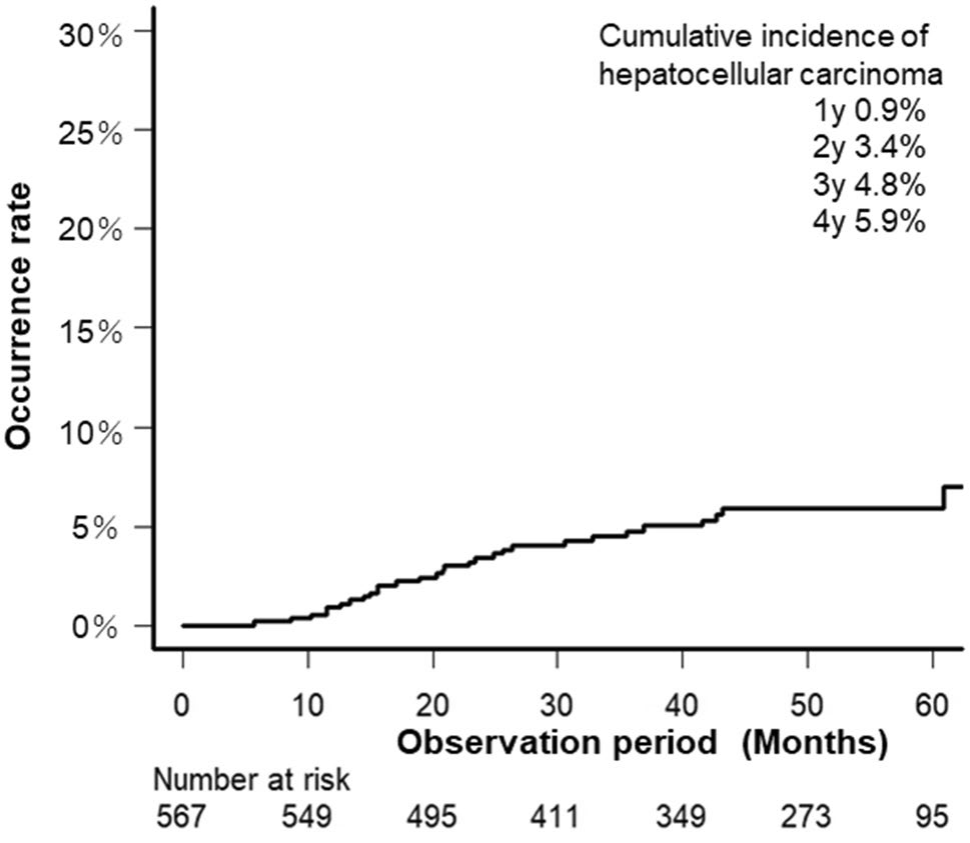
Cumulative incidence of hepatocellular carcinoma after IFN-free treatment. IFN, interferon.

During the observation period, 30 (5.3%) patients developed HCC. The cumulative incidence rates of HCC after successful HCV eradication were 0.9% for 1 year, 3.4% for 2 years, 4.8% for 3 years, and 5.9% for 4 years (Fig. 1).

Among 30 HCC occurrence cases, 4 patients (8.3%) were initially found by an increase in AFP, and the remaining cases were initially found by imaging surveillance. Curative treatment (liver surgery or radio frequency ablation) was performed on 25 cases (83.3%).

### Change in LSM after hepatitis C virus (HCV) eradication

**Figure 2:**
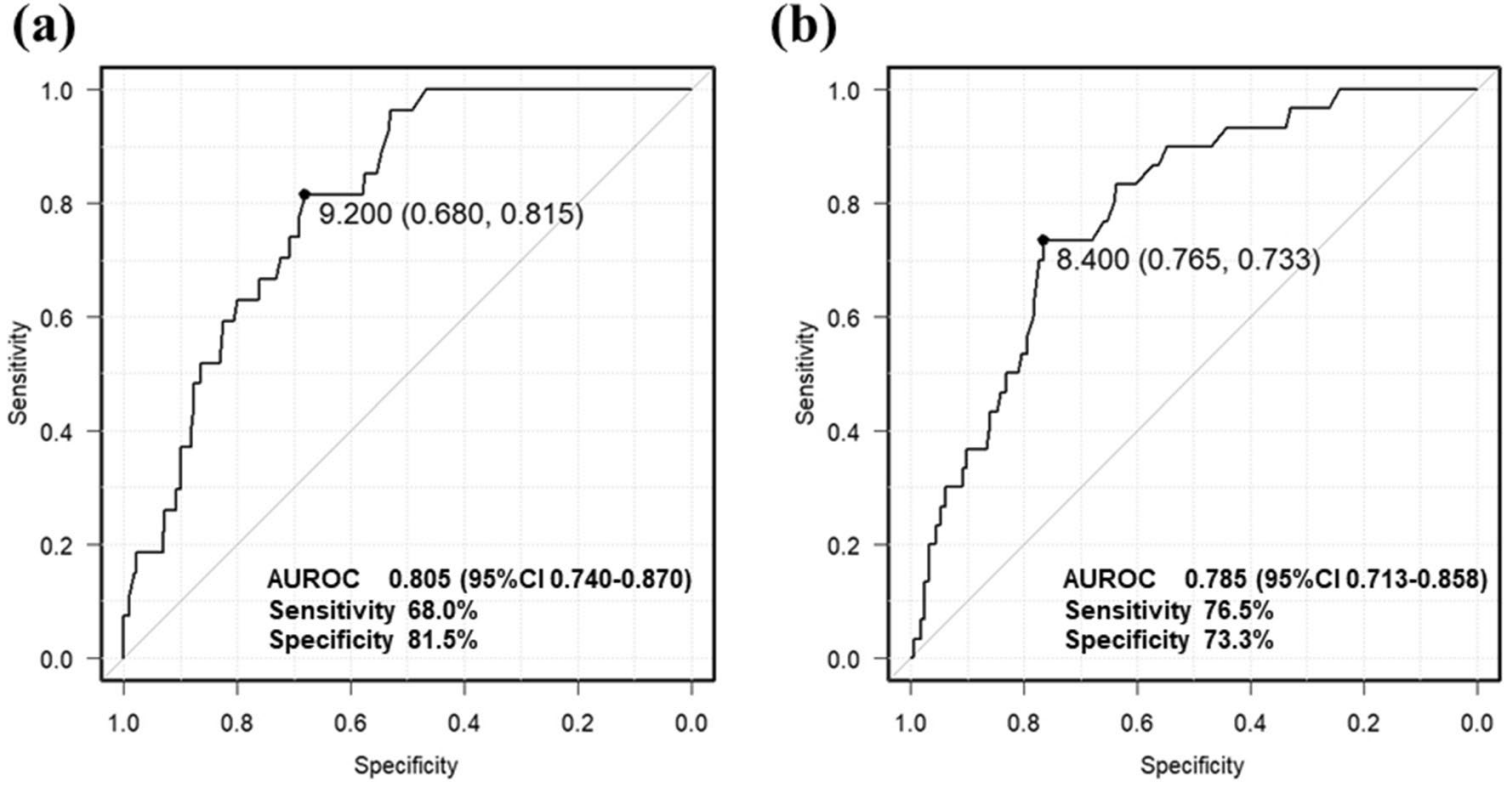
ROC curve analysis of LSM and prediction of HCC. The ROC curve shows LSM in predicting HCC after SVR with IFN-free treatment. (**a**) Pre-treatment LSM. (**b**) LSM at the time of SVR24. ROC, receiver operating characteristic; LSM, liver stiffness measurement; HCC, hepatocellular carcinoma; SVR24, sustained virological response at 24 weeks.

We investigated the changes in liver stiffness before and after HCV eradication. Pre-treatment LSM data were available for 521 out of the 567 patients. Although the median liver stiffness before HCV treatment was 7.1 kPa, that at time of SVR24 was 5.6 kPa—a significant decrease of 1.5 kPa with treatment (*p* < 0.01, Supplemental Fig. 1). Additionally, ALT decreased from 32 to 15 IU/L, AFP from 4.2 to 3.2 ng/mL, and FIB4 index from 2.72 to 2.14. ROC curve analysis was used to examine the best cut-off value for the occurrence of HCC according to pre-treatment and post-treatment liver stiffness. The results revealed that the pre-treatment cut-off value was 9.2 kPa (sensitivity, 68%; specificity, 81.5%), and the cut-off value at the time of SVR24 was 8.4 kPa (sensitivity, 76.5%; specificity, 73.3%) (Fig. 2).

### Factors associated with HCC after HCV eradication

Subsequently, we examined the factors associated with HCC occurrence after HCV eradication. In order to predict HCC occurrence using ROC curve analysis, the optimal cut-off age value was calculated as 71 years old (sensitivity, 65.7%; specificity, 60.0%; Supplemental Fig. 3). Similarly, optimal cut-off values of blood data at the start of treatment and at SVR24 were calculated by ROC curve analysis and used for the following analysis.

**Figure 3:**
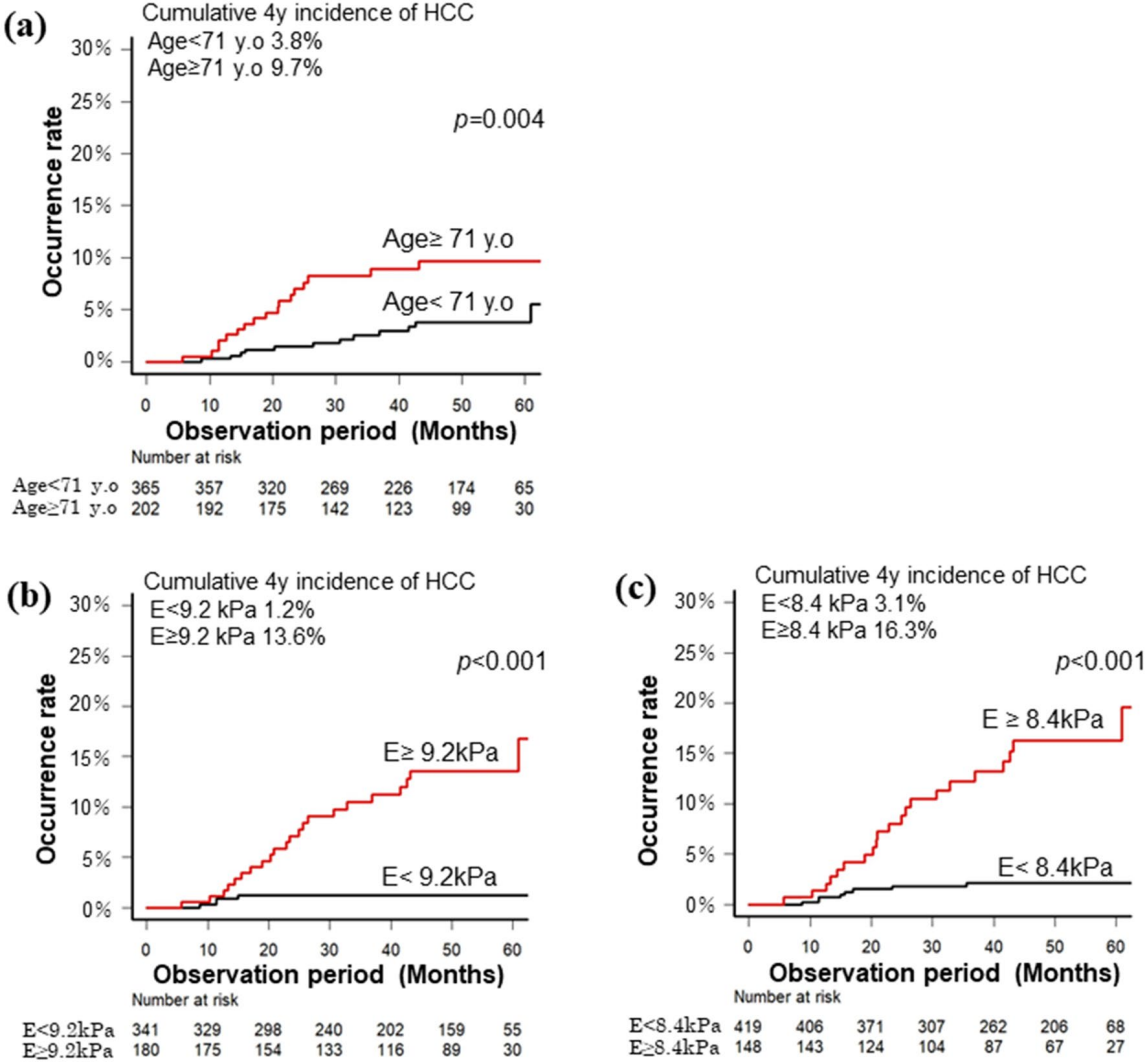
Cumulative incidence of hepatocellular carcinoma after IFN-free treatment according to factors contributing to HCC. Cumulative incidence of hepatocellular carcinoma according to (**a**) age of 71 years as a cut-off value. The black line indicates patients younger than 71 years, and the red line indicates patients aged > 71 years. (**b**) LSM before treatment with 9.2 kPa as the cut-off value. The black line indicates cases with LSM < 9.2 kPa, and the red line indicates those with LSM > 9.2 kPa. (**c**) LSM at the time of SVR24 with 8.4 kPa as the cut-off value. The black line indicates cases with LSM < 8.4 kPa, and the red line indicates those with LSM > 8.4 kPa. IFN, interferon; HCC, hepatocellular carcinoma; LSM, liver stiffness measurement; SVR24, sustained virological response at 24 weeks.

First, pre-treatment univariate analysis revealed that age ≥ 71 years, diabetes, platelet count < 13.2 × 10^4^/µL, albumin < 3.9 g/dL, AFP ≥ 6.2 ng/mL, FIB 4 index ≥ 3.25, and LSM ≥ 9.2 kPa were significant contributors of HCC occurrence. Platelet count is a confounding factor for FIB4; therefore, after excluding it, multivariate analysis revealed that age ≥ 71 years (*p* = 0.005) and liver stiffness ≥ 9.2 kPa (*p* < 0.001) were significantly associated with HCC occurrence after SVR24 (Table 2 and Fig. 3). Pre-treatment AFP was not significantly different from post-treatment AFP; however, it tended to be associated with HCC occurrence (*p* = 0.062).

**Table 2:** Predictive factors at pre-treatment for occurrence of HCC after SVR.

Factors	Cut-off value	Univariate *p* values	Multivariate		
			HR	95% CI	*p*-values
Sex	Male	0.185			
Age (years old)	≥ 71	**0.004**	**3.402**	**1.447–8.001**	**0.005**
BMI (kg/m^2^)	≥ 25	0.097			
HCV genotype	1	0.289			
Diabetes	Yes	**0.029**	1.479	0.653–3.351	0.349
Therapy resume; SOF based	Yes	0.206			
Pre platelet (× 10^4^/µl)	≥ 13.2	**0.004**	–		
Pre ALT (IU/L)	≥ 40	0.810			
Pre albumin (g/dl)	≥ 3.9	**0.011**	1.419	0.621–3.245	0.407
Pre AFP (ng/ml)	≥ 6.2	**0.002**	2.400	0.957–6.017	0.062
Pre FIB4 index	≥ 3.25	**0.023**	0.541	0.201–1.460	0.296
Pre LSM (kPa)	≥ 9.2	** < 0.001**	**6.328**	**2.205–18.160**	** < 0.001**
Pre CAP (db/m)	≥ 230	0.106			

*BMI* Body mass index, *HCV* Hepatitis C virus, *SOF* Sofosbuvir, *Pre* Pre-treatment, *AST* Aspartate transaminase, *ALT* Alanine aminotransferase, *GGT* γ-glutamyltransferase, *AFP* α-fetoprotein, *LSM* Liver stiffness measurement, *CAP* Controlled attenuation parameter.

Similarly, factors at the time of SVR24 related to HCC occurrence were examined. In the univariate analysis, age ≥ 71 years, diabetes, platelet count < 12.0 × 10^4^/µL, AFP ≥ 4.2 ng/mL, FIB4 index ≥ 2.73, and LSM ≥ 8.4 kPa were identified as factors significantly associated with HCC after SVR. However, on multivariate analysis, age ≥ 71 years (*p* = 0.014) and LSM ≥ 8.4 kPa (*p* < 0.001) were significant contributors of HCC occurrence in the multivariate analysis (Table 3 and Fig. 3).

**Table 3:** Predictive factors at SVR24 for occurrence of HCC after SVR.

Factors	Cut-off value	Univariate *p* values	Multivariate		
			HR	95% CI	*p*-values
Sex	Male	0.185			
Age (years old)	≥ 71	**0.004**	**2.689**	**1.222–5.914**	**0.014**
BMI (kg/m^2^)	≥ 25	0.097			
HCV genotype	1	0.289			
Diabetes	Yes	**0.029**	1.673	0.767–3.648	0.196
Therapy resume; SOF based	Yes	0.206			
SVR24 Platelet (× 10^4^/µl)	≥ 12.0	**0.045**	–		
SVR24 ALT (IU/L)	≥ 40	0.380			
SVR24 Alb (g/dL)	≥ 4.3	0.577			
SVR24 AFP (ng/ml)	≥ 4.2	** < 0.001**	1.683	0.739–3.833	0.216
SVR24 FIB4 index	≥ 2.73	**0.008**	0.983	0.426–2.270	0.968
SVR24 LSM (kPa)	≥ 8.4	** < 0.001**	**6.642**	**2.527–17.46**	** < 0.001**
SVR24 CAP (db/m)	≥ 230	0.451			

*BMI* Body mass index, *HCV* Hepatitis C virus, *SOF* Sofosbuvir, *SVR24* Sustained viral response at 24 weeks, *AST* Aspartate transaminase, *ALT* Alanine aminotransferase, *GGT* γ-glutamyltransferase, *AFP* α-fetoprotein, *LSM* Liver stiffness measurement, *CAP* Controlled attenuation parameter.

Even in the analysis limited to elderly patients (age ≥ 71 years; 202 patients), only LSM ≥ 9.2 kPa at pre-treatment and LSM ≥ 8.4 kPa at SVR24 were the factors that significantly predicted HCC occurrence after SVR in multivariate analysis (Supplemental Tables 1 and 2).

### High-risk groups for HCC occurrence using LSM and age

**Figure 4:**
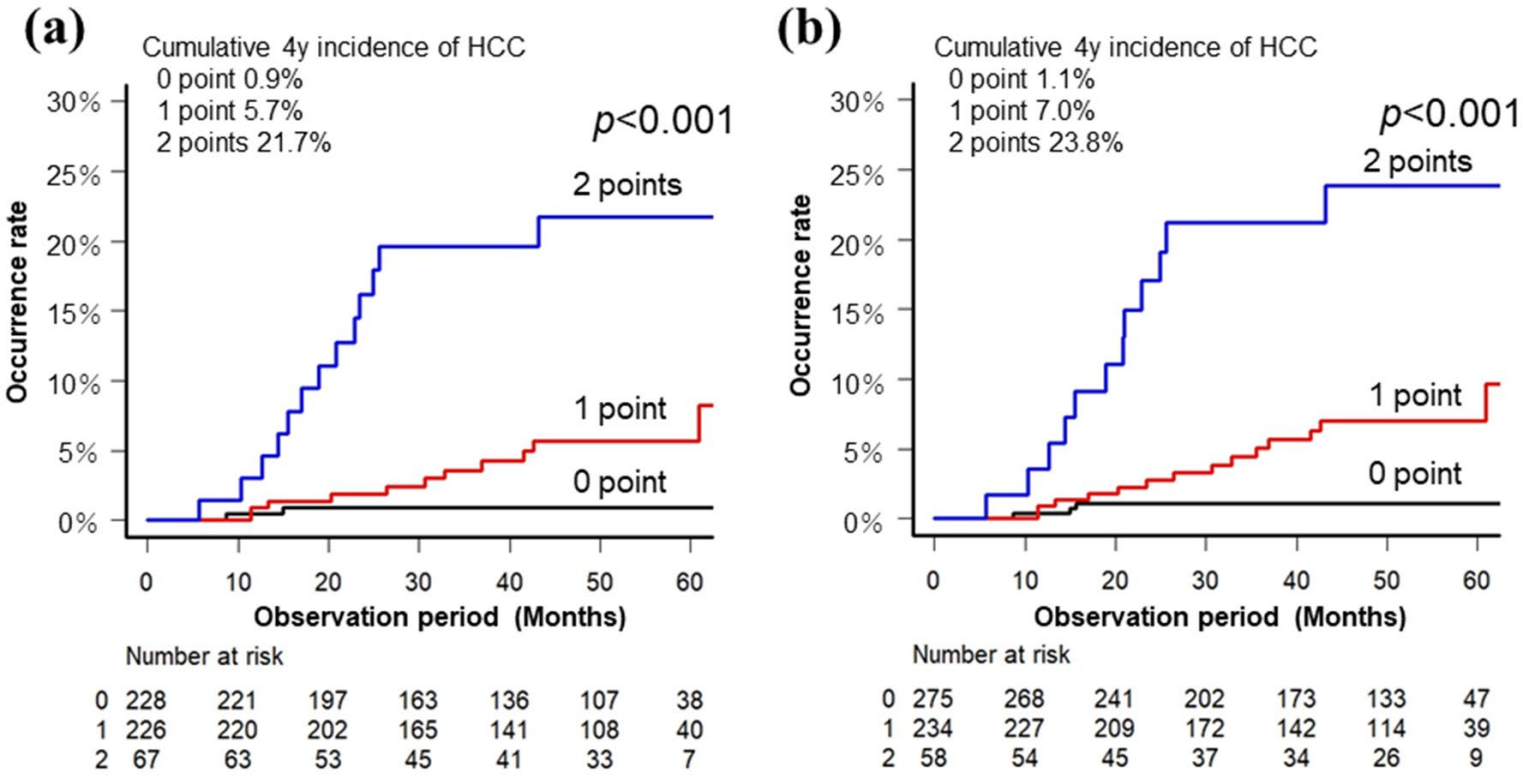
Identification of high-risk and low-risk groups of HCC due to age and LSM. Cumulative incidence of HCC according to age and LSM divided into three groups. When the age and LSM were above the cut-off values, 1 point was assigned to each and divided into three groups of 0–2 points. The black line indicates 0-point cases, red line indicates 1-point cases, and blue line indicates 2-point cases. (**a**) When grouped according to age and LSM pre-treatment, the age was 71 years, and the cut-off value for LSM was 9.2 kPa. (**b**) When grouped according to age and LSM at the time of SVR24, the age was 71 years, and the cut-off value for LSM was 8.4 kPa. HCC, hepatocellular carcinoma; LSM, liver stiffness measurement; SVR24, sustained virological response at 24 weeks.

We investigated whether it is possible to identify high-risk and low-risk groups of HCC after SVR using two factors, age and liver stiffness, which were significant in the multivariate analysis. Before treatment, 1 point each was assigned for age ≥ 71 years and LSM ≥ 9.2 kPa, and the total points were divided into three groups (0, 1, and 2 points) for analysis. Similarly, at the time of SVR24, we assigned 1 point each to age ≥ 71 years and LSM ≥ 8.4 kPa. Subsequently, they were analyzed by dividing them into three groups. As shown in Fig. 4, based on both pre-treatment factors, the 4-year cumulative incidence rates of HCC (0, 1, and 2 points) in the three groups were 0.9%, 5.7%, and 21.7% (*p* < 0.001), respectively, whereas based on factors at the time of SVR24, the rates were 1.1%, 7.0%, and 23.8% (*p* < 0.001), respectively. Stratification was possible using either factor. Notably, no HCC occurrence was observed in the 0-point population after 1 year of treatment completion.

## Discussion

In this multicenter retrospective study, we analyzed the predictive factors associated with the occurrence of HCC after HCV eradication using IFN-free DAAs. Our findings revealed that advanced age and LSM on transient elastography before treatment and at the time of SVR24 are important predictors of HCC occurrence. Additionally, based on these two non-invasive factors, we could identify patients at high and low risk of HCC after successful HCV eradication with DAAs.

The progression of liver fibrosis diagnosed using liver histology has been widely reported as an excellent predictor of HCC occurrence after IFN-based treatment^3–15,17^ and IFN-free treatment^18^. In recent years, as a substitute for histological evaluation of liver fibrosis, LSM using ultrasound or magnetic resonance elastography has become possible, which is considered a useful evaluation method for liver fibrosis. According to the European Association for the Study of the Liver (EASL) guidelines, it is not necessary to perform liver biopsy only for the diagnosis of the stage of liver fibrosis. Therefore, non-invasive methods such as elastography are recommended^19^.

Elastography is a method of evaluating changes in tissues using ultrasonic waves or MRI due to mechanical compression from outside the body or acoustic radiation force impulse within the device. LSM has been demonstrated to correlate with histological liver fibrosis in untreated patients with chronic hepatitis C^20–22^ and various liver diseases^21,23–25^. Correlation between histological fibrosis and cut-off values for fibrosis stages were reported for each method. Ultrasonic elastography has the advantage of being more versatile and relatively easy than MRI elastography. In this study, we analyzed a large number of cases using transient elastography, which is considered easier to use in daily clinical practice.

In hepatitis C, there is inflammation in the liver due to HCV infection. It is known that inflammation results in high serum levels of ALT and fibrosis markers, and antiviral treatments lower these levels. Elastography is also known to be affected by inflammation, and the EASL-Latin American Association for the Study of the Liver clinical practice guidelines state that LSM is high when ALT is ≥ five times the upper limit of normal in patients with hepatitis B infection^26^. As shown in Supplemental Fig. 1, the LSM level also decreased significantly after IFN-free treatment in this study, which reflects the improvement in inflammation in the liver. We previously reported the usefulness of LSM in various liver diseases and that HCC incidence was significantly higher with LSM > 8.0 kPa in HCV^27^. Wang et al^13^ have also reported the importance of LSM as a risk factor for HCC occurrence after IFN treatment. However, their report was an analysis of a young cohort (median age, 54 years) with a median period between SVR and LSM measurement of 35 months, which is not consistent with HCV eradication.

To date, few studies have reported the usefulness of LSM in predicting the occurrence of HCC after DAAs; however, the median age in those studies was relatively younger than that in our study, and the analyses were limited to patients with advanced chronic liver disease. Pons et al^28^ have reported the usefulness of LSM in 572 cases of compensated advanced chronic liver disease (cACLD) according to the Baveno VI criteria^29^. They reported that 25 cases of HCC occurrence were observed in the 2.8-year observation period, and that serum albumin and LSM values were reported as useful predictors of HCC occurrence after SVR. Similarly, Alonso et al^30^ have analyzed 993 cases of cACLD for > 45 months. They reported 32 cases of HCC and that LSM, serum albumin at the end of treatment, rate of change in LSM after the end of treatment, and FIB4 index after 1 year were significant predictors of HCC occurrence after SVR. Based on these reports, LSM is a useful predictor of HCC occurrence in cACLD. However, as described before, the cohorts of these two reports were limited to cACLD and a lower median age (63.7 and 61.7 years, respectively). Notably, since the cohort of this study included many cases of non-advanced chronic liver disease (68.7%) and elderly patients (median age, 66.0 years; those of 71 years of age or older, 35.6%), it is considered that the analysis targets were different from those in our study. In contrast to these two studies, in addition to liver fibrosis, multivariate analysis in our study revealed that advanced age (≥ 71 years) was significantly associated with the occurrence of HCC after DAAs. Advanced age has been reported to be an important factor in HCC occurrence after HCV treatment with IFN-based therapy^3–12,14,17,31–39^. Importantly, when analyzed in only cases with LSM < 8.4 kPa at the time of SVR24, the 4-year cumulative HCC incidence rate tended to be higher in patients aged ≥ 71 years (< 71 years: 1.1%, ≥ 71 years: 4.1%, Supplemental Fig. 2). Therefore, even in cases not limited to those with advanced fibrosis, LSM is an important predictor of HCC occurrence after HCV eradication following DAAs, and advanced age is an important predictor of HCC occurrence after SVR in the general population.

Regarding MRI elastography, Higuchi et al. have reported that cases with magnetic liver elasticity ≥ 3.75 kPa at SVR12 were at a high risk of HCC during the 21-month observation period^40^. Based on these reports, LSM is useful as a predictor of HCC occurrence after HCV eradication. However, it is desirable to standardize the timepoints for the evaluation of LSM and analyze them over a longer observation period. In this study, we analyzed cases in which LSM could be measured at the time of SVR24, which is generally the goal of anti-HCV treatment of approximately 50 months. As a result of ROC analysis for predicting HCC occurrence, the cut-off values of LSM were 9.2 kPa before treatment and 8.4 kPa at the time of SVR24. Pre-treatment LSM and LSM at the time of SVR24 were factors that significantly predicted HCC occurrence after SVR in the multivariate analysis.

So far, various predictors for HCC occurrence after SVR by IFN-free DAAs therapy, based on the factors evaluated before or after treatment, have been reported. The usefulness of FIB4 index^41^, Wisteria floribunda agglutinin-positive Mac-2 binding protein (M2BPGi)^42^ and total bilirubin^38^ as pre-treatment factors, and FIB4 index^43^, AFP^18,41,42,44^ and M2BPGi^42,43,45^ as post-treatment factors for predicting HCC occurrence after SVR has been reported. In a report which analyzed LSM values before and after treatment, LSM values at 1 year after end of treatment were a statistically significant factor predicting HCC after SVR^28^. Post-treatment LSM values may be a better predictor than pre-treatment values. However, no conclusion has been reached as to which of the pre-treatment and post-treatment factors is more useful as a predictor of HCC after SVR.

In this study, LSM at both pre-treatment and at the time of SVR24 demonstrated good area under the curve (0.805 and 0.785, respectively; Fig. 2) for predicting HCC after SVR. Therefore, even if LSM can be measured only at either pre-treatment or at the time of SVR24, it may be possible to predict the risk of HCC using the appropriate cut-off values for the respective period.

Recent studies demonstrated that FIB4 index are associated with HCC after SVR^41,46^. FIB4 index can be easily and repeatedly calculated only by age, AST, ALT and platelet count. However, FIB4 index may overestimate the degree of liver fibrosis, as it can be higher in elderly patients. A report examining the usefulness of FIB4 index for predicting high LSM levels in NAFLD patients presented the need for higher cut-off FIB4 index in older patients^47^. In this study, we also analyzed 202 patients with age ≥ 71 years. As shown in Supplemental Tables 1 ans 2, the FIB4 index was not a significant predictor of HCC after SVR even in univariate analysis in the group of patients with age ≥ 71 years. Therefore, LSM might more accurately reflect liver fibrosis than FIB4 index, and may be useful in predicting HCC occurrence after SVR, especially in elderly patients.

M2BPGi, a novel fibrosis marker of HCV, has been also recently reported as a predictor of HCC occurrence after SVR^36,42–45^. M2BPGi can also be repeatedly tested using only blood. We also analyzed HCC occurrence after SVR in patients in which M2BPGi data was available (431 cases, including HCC occurrence in 18 cases). In this cohort, M2BPGi was not a significant factor in multivariate analysis, but LSM was a significant predictor of HCC occurrence (Supplemental Tables 3 and 4). M2BPGi might reflect other factors, such as liver inflammation, because it was reported that M2BPGi increases after acute liver injury^48^.

However, because the included number of patients were limited in this analysis, further analysis is required.

In contrast, diabetes has been reported to contribute to HCC in IFN-based therapy^8,13,31,38,49–51^. Furthermore, diabetes is considered a risk factor for HCC even in cases without HCV, and it has been reported that HR of HCC incidence is approximately 2.5 times higher in patients with diabetes^52^. However, in this study, diabetes was a significant factor of HCC only in the univariate analysis, not in the multivariate analysis. In this study, the median observation period was approximately 50 months, and only relatively early HCC following HCV eradication was observed. Therefore, diabetes may not have been a significant contributor of the occurrence of HCC. Evaluations over longer observation periods suggest that diabetes may be an independent contributor of HCC in addition to LSM and advanced age.

Since eradication of HCV is now possible in almost all cases, HCC surveillance after SVR has become a clinically important issue. Since each case has a different risk of HCC occurrence, an appropriate follow-up interval after HCV eradication has not been determined. From the perspective of health economics, tight follow-up is desirable for those at a high risk of HCC, whereas longer follow-up intervals are ideal for those at a low risk of HCC. In this study, we also focused on whether it was possible to identify a low-risk group regarding HCC after SVR. As illustrated in Fig. 4, the 4-year cumulative HCC rate was extremely low, 1.1%, in those aged < 71 years and with LSM < 8.4 kPa at the time of SVR24. Furthermore, HCC in these cases was observed within 16 months after the start of treatment and was not observed thereafter. Therefore, regarding post-SVR follow-up of HCV cases with no history of HCC, patients younger than 71 years with lower LSM (< 8.4 kPa at the time of SVR24) have a significantly low risk of HCC at 1 year after SVR24. Therefore, it is possible to increase the follow-up interval for such patients.

The limitations of our study need to be acknowledged. First, this was a retrospective observational study with a median observation period of approximately 4 years. Additionally, several data, including serum fibrosis markers, were lacking. A prospective study with more data and longer observation periods is necessary. Second, this analysis excluded cases in which LSM at the time of SVR24 was difficult to measure. Therefore, the absence of patients with severe obesity may be biased. Third, serum fibrosis markers such as M2BPGi, hyaluronic acid, and Type IV collagen 7S have many missing values, and analysis using these markers was not possible. Fourth, we have not been able to verify the results of this study using a validation cohort. In the future, it would be desirable to validate the results of this study in another cohort.

In conclusion, advanced age and progression of liver fibrosis on transient elastography are important risk factors for HCC following SVR with IFN-free treatment. Furthermore, it was suggested that the use of these two factors may help identify the low-risk group for HCC. Our results could be used in HCC surveillance following SVR.

## Methods

### Patients and surveillance of HCC

Overall, 567 consecutive patients with HCV treated with DAAs who had achieved SVR at 24 weeks (SVR24) between September 2015 and October 2020 at the Hokkaido University Hospital, Hakodate Municipal Hospital, and JCHO Hokkaido Hospital were retrospectively analyzed. The exclusion criteria were history of HCC before SVR24, follow-up ≤ 6 months after the end of treatment, insufficient pre-DAA and post-DAA evaluation of the occurrence of HCC, and no LSM data at the time of SVR24. HCC surveillance was performed using imaging modalities, such as ultrasonography, computed tomography, or magnetic resonance imaging (MRI), and tumor markers (alpha-fetoprotein [AFP], des-gamma-carboxy prothrombin, and lens culinaris agglutinin-reactive fraction of AFP) before the initiation of treatment and subsequently every 3–6 months were also evaluated. The definition of cirrhosis was based on the following findings: (1) unequivocal ultrasound signs of cirrhosis (blunted nodular liver surface and/or splenomegaly), (2) development of portosystemic shunts such as gastroesophageal varices, and (3) histologic diagnosis of F4 fibrosis.

### Measurement of liver stiffness

To measure LSM and controlled attenuation parameter, FibroScan 502 (Echosens, Paris, France) using the M probe or XL probe was utilized. Imaging was performed with the patients in the supine position and right arm at the most abducted position for right intercostal imaging. Effective measurements were defined as at least 10 measurements with effective measurement ≥ 60% and interquartile range < 30%, and the median of the values was included in the analyses.

### Ethical considerations

The study protocol was approved by the Institutional Ethics Committee of Hokkaido University Hospital, Hakodate Municipal Hospital, and JCHO Hokkaido Hospital (Approval Protocol No. 016-0021). The study conformed to the ethical guidelines of the Declaration of Helsinki. Informed consent was obtained from all patients.

### Statistical analyses

Statistical analyses were performed using EZR software^53^. The Mann–Whitney U test was used to compare the continuous data between the two groups. Fisher’s exact test was used for univariate analysis of ordered variables. The optimal cut-off values for the univariate and multivariate analyses were set to yield the largest Youden index on the receiver operating characteristic (ROC) curve analysis^54,55^. The cumulative incidence of HCC was calculated using the Kaplan–Meier method, and the *p*-value for group comparisons was obtained using the log-rank test. The Cox proportional hazards model was used for multivariate analysis of the cumulative incidence of HCC. *P* < 0.05 was considered statistically significant.

## Supplementary Information

Supplementary Information 1.

Supplementary Information 2.
