# Supplementary material for: Prediction of hepatocellular carcinoma using age and liver stiffness on transient elastography after hepatitis C virus eradication
Source: Sci Rep. 2022 Jan 27;12:1449. doi: 10.1038/s41598-022-05492-5 (PMC8795443; doi:10.1038/s41598-022-05492-5)
Supplement: Supplementary file 1 — Supplementary Information 1. [file 41598_2022_5492_MOESM1_ESM.pdf]

Supplemental Figure 1 Liver stiffness measurement (LSM) before HCV treatment and at the time of SVR24

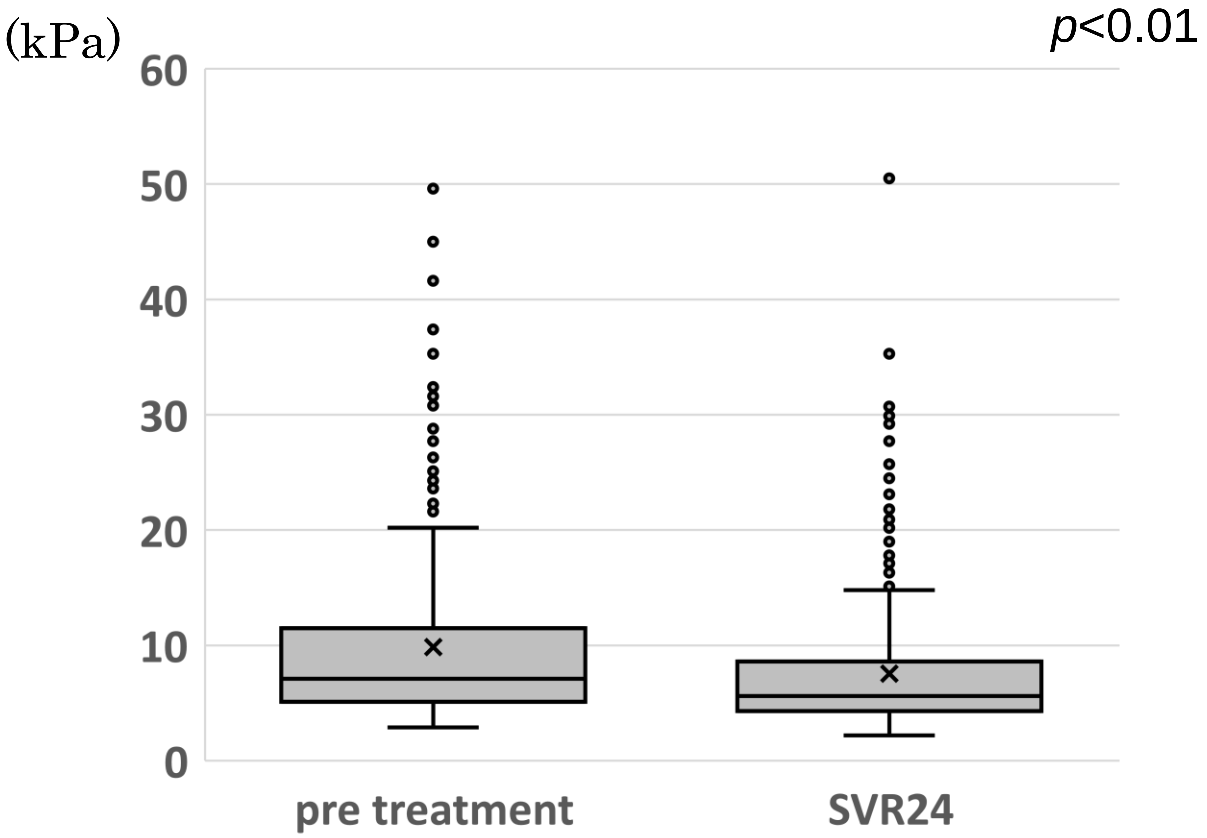

In the box charts for the Y-axis, the bold lines indicate the median, “x” indicates the average, boxes indicate the 25th and 75th percentiles, and lines for each edge indicate the 10th and 90th percentiles.

HCV, hepatitis C; *SVR24*, sustained virological response at 24 weeks.

Supplemental Figure 2 Cumulative incidence of hepatocellular carcinoma after IFN-free treatment in patients with low LSM divided by age

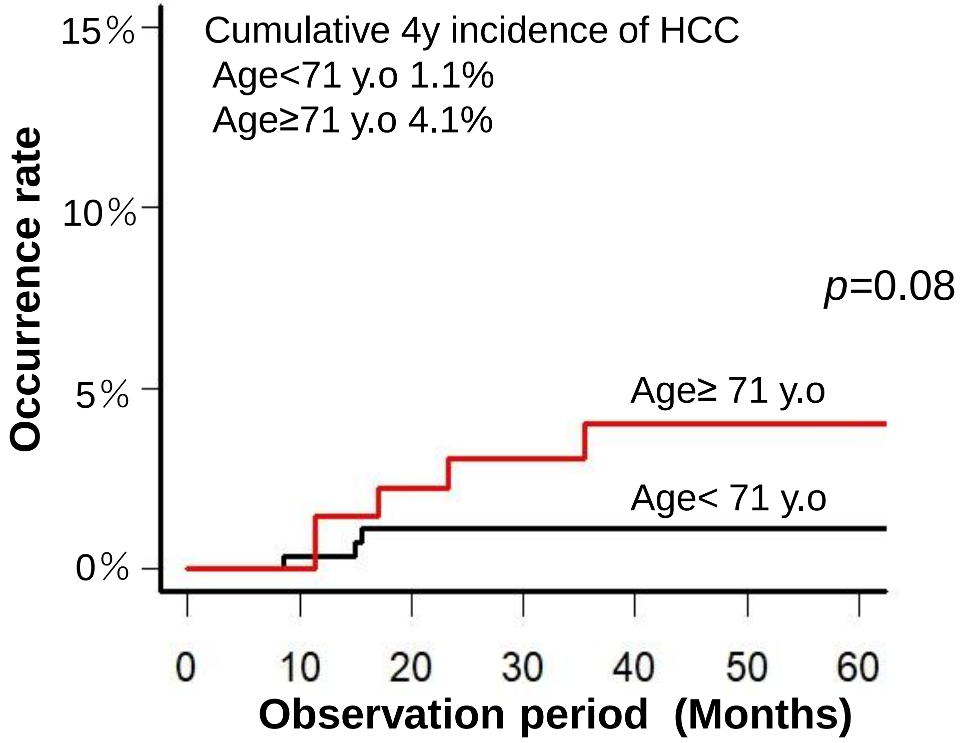

Number at risk

**Age<71 y.o**	275	268	241	202	173	133	47
**Age≥71 y.o**	143	137	129	104	88	72	20

Cumulative incidence of hepatocellular carcinoma in patients with LSM <8.4 kPa at the time of SVR24 divided according to age of 71 years as the cut-off value. The black line indicates those younger than 71 years, and the red line indicates those older than 71 years.

IFN, interferon; *HCC*, hepatocellular carcinoma; *LSM*, liver stiffness measurement; *SVR24*, sustained virological response at 24 weeks

Supplemental Figure 3 ROC curve analysis of age and prediction of HCC

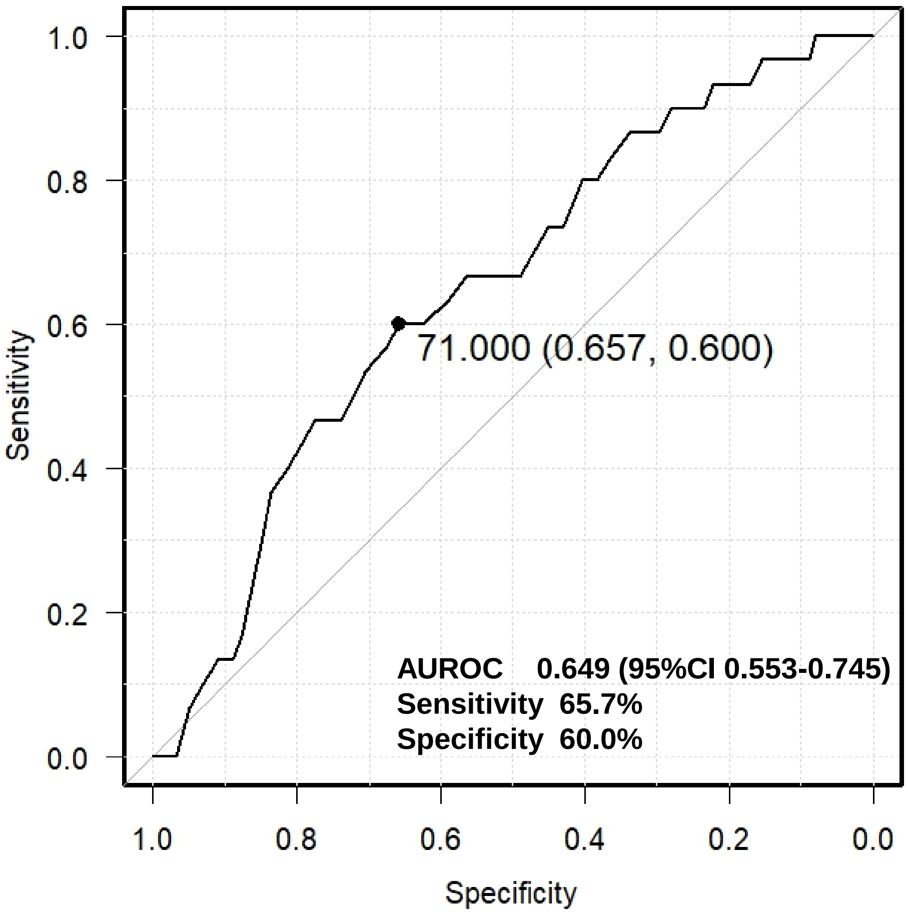

The ROC curve showing age as a predictor of HCC after SVR with IFN-free treatment.
ROC, receiver operating characteristic; *HCC*, hepatocellular carcinoma
