# Supplementary material for: Prediction of hepatocellular carcinoma using age and liver stiffness on transient elastography after hepatitis C virus eradication
Source: Sci Rep. 2022 Jan 27;12:1449. doi: 10.1038/s41598-022-05492-5 (PMC8795443; doi:10.1038/s41598-022-05492-5)
Supplement: Supplementary file 2 — Supplementary Information 2. [file 41598_2022_5492_MOESM2_ESM.docx]

*Supplemental Table 1. Predictive factors at pre-treatment for Occurrence of HCC after SVR in patients with age≥71 years old*

| Factors | Cut-off value | Univariate  *p* values | Multivariate  HR 　 　95%CI 　　 *p*-values | | |
| --- | --- | --- | --- | --- | --- |
| Sex | Male | 0.067 |  |  |  |
| BMI (kg/m^2^) | ≥25 | 0.224 |  |  |  |
| HCV Genotype | 1 | 0.678 |  |  |  |
| Diabetes | Yes | 0.073 |  |  |  |
| Therapy resume; SOF based | Yes | 0.365 |  |  |  |
| Pretreatment Platelet (×10^4^/µl) | ≤13.2 | 0.144 | - |  |  |
| Pretreatment ALT (IU/L) | ≥40 | 0.766 |  |  |  |
| Pretreatment Albumin (g/dl) | ≤3.9 | 0.486 |  |  |  |
| Pretreatment AFP (ng/ml) | ≥6.2 | **0.027** | 1.830 | 0.654-5.115 | 0.249 |
| Pretreatment FIB4 index | ≥3.25 | 0.617 |  |  |  |
| Pretreatment LSM (kPa) | ≥9.2 | **<0.001** | **6.422** | **1.736-23.76** | **0.006** |
| Pretreatment CAP (db/m) | ≥230 | 0.614 |  |  |  |

*BMI* Body mass index, *HCV* Hepatitis C virus, *AST* Aspartate transaminase, *ALT* Alanine aminotransferase, *GGT* γ-glutamyltransferase, *AFP* α-fetoprotein, *LSM* Liver stiffness measurement, *CAP* Controlled attenuation Parameter

*Supplemental Table 2. Predictive factors at SVR24 for Occurrence of HCC after SVR in patients with age≥71 years old*

| Factors | Cut-off value | Univariate  *p* values | Multivariate  HR 　95%CI 　 *p*-values | | |
| --- | --- | --- | --- | --- | --- |
| Sex | Male | 0.067 |  |  |  |
| BMI (kg/m^2^) | ≥25 | 0.224 |  |  |  |
| HCV Genotype | 1 | 0.678 |  |  |  |
| Diabetes | Yes | 0.073 |  |  |  |
| Therapy resume; SOF based | Yes | 0.365 |  |  |  |
| SVR24 Platelet (×10^4^/µl) | ≤12.0 | 0.175 | - |  |  |
| SVR24 ALT (IU/L) | ≥40 | 0.631 |  |  |  |
| SVR24 Alb (g/dL) | ≤4.3 | 0.723 |  |  |  |
| SVR24 AFP (ng/ml) | ≥4.2 | **0.003** | 1.827 | 0.639-5.214 | 0.261 |
| SVR24 FIB4 index | ≥2.73 | 0.359 |  |  |  |
| SVR24 LSM (kPa) | ≥8.4 | **<0.001** | **7.029** | **2.094-23.60** | **0.002** |
| SVR24 CAP (db/m) | ≥230 | 0.845 |  |  |  |

*BMI* Body mass index, *HCV* Hepatitis C virus, *AST* Aspartate transaminase, *ALT* Alanine aminotransferase, *GGT* γ-glutamyltransferase, *AFP* α-fetoprotein, *LSM* Liver stiffness measurement, *CAP* Controlled attenuation Parameter

*Supplemental Table 3. Predictive factors at pre-treatment for Occurrence of HCC after SVR in patients with M2BPGi data available*

| Factors | Cut-off value | Univariate  *p* values | Multivariate  HR 　　95%CI 　　 *p*-values | | |
| --- | --- | --- | --- | --- | --- |
| Sex | Male | 0.186 |  |  |  |
| Age (years old) | ≥71 | 0.076 |  |  |  |
| BMI (kg/m^2^) | ≥25 | 0.091 |  |  |  |
| HCV Genotype | 1 | 0.530 |  |  |  |
| Diabetes | Yes | 0.216 |  |  |  |
| Therapy resume; SOF based | Yes | 0.524 |  |  |  |
| Pretreatment Platelet (×10^4^/µl) | ≤13.2 | **<0.001** | - |  |  |
| Pretreatment ALT (IU/L) | ≥40 | 0.604 |  |  |  |
| Pretreatment Albumin (g/dl) | ≤3.9 | **0.003** | 2.720 | 0.800-9.247 | 0.234 |
| Pretreatment AFP (ng/ml) | ≥6.2 | **0.002** | 2.878 | 0.817-10.140 | 0.099 |
| Pretreatment FIB4 index | ≥3.25 | **0.003** | 1.942 | 0.388-9.711 | 0.418 |
| Pretreatment LSM (kPa) | ≥9.2 | **<0.001** | **17.20** | **2.084-142.10** | **0.009** |
| Pretreatment CAP (db/m) | ≥230 | 0.573 |  |  |  |
| Pretreatment M2BPGi (C.O.I) | ≥2.50 | **0.004** | 1.904 | 0.449-8.077 | 0.383 |

*BMI* Body mass index, *HCV* Hepatitis C virus, *AST* Aspartate transaminase, *ALT* Alanine aminotransferase, *GGT* γ-glutamyltransferase, *AFP* α-fetoprotein, *LSM* Liver stiffness measurement, *CAP* Controlled attenuation Parameter

*Supplemental Table 4. Predictive factors at SVR24 for Occurrence of HCC after SVR in patients with M2BPGi data available*

| Factors | Cut-off value | Univariate  *p* values | Multivariate  HR 　 95%CI 　 *p*-values | | |
| --- | --- | --- | --- | --- | --- |
| Sex | Male | 0.186 |  |  |  |
| Age (years old) | ≥71 | 0.076 |  |  |  |
| BMI (kg/m^2^) | ≥25 | 0.091 |  |  |  |
| HCV Genotype | 1 | 0.530 |  |  |  |
| Diabetes | Yes | 0.216 |  |  |  |
| Therapy resume; SOF based | Yes | 0.524 |  |  |  |
| SVR24 Platelet (×10^4^/µl) | ≤12.0 | **0.002** | - |  |  |
| SVR24 ALT (IU/L) | ≥40 | 0.524 |  |  |  |
| SVR24 Alb (g/dL) | ≤4.3 | 0.533 |  |  |  |
| SVR24 AFP (ng/ml) | ≥4.2 | **0.008** | 1.641 | 0.504-5.343 | 0.411 |
| SVR24 FIB4 index | ≥2.73 | **<0.001** | 3.224 | 0.737-14.10 | 0.061 |
| SVR24 LSM (kPa) | ≥8.4 | **<0.001** | **13.45** | **2.605-69.42** | **0.002** |
| SVR24 CAP (db/m) | ≥230 | 0.446 |  |  |  |
| SVR24 M2BPGi (C.O.I) | ≥1.37 | **0.011** | 1.891 | 0.527-6.778 | 0.328 |

*BMI* Body mass index, *HCV* Hepatitis C virus, *AST* Aspartate transaminase, *ALT* Alanine aminotransferase, *GGT* γ-glutamyltransferase, *AFP* α-fetoprotein, *LSM* Liver stiffness measurement, *CAP* Controlled attenuation Parameter
